# Patients' Views on Medical Events in Lung Cancer Screening as Teachable Moments for Smoking Behaviour Change: A Systematic Review and Metasynthesis

**DOI:** 10.1155/2023/6647364

**Published:** 2023-06-03

**Authors:** Anvita Vikram, Claire Muller, Lucy Hulme

**Affiliations:** ^1^Pennine Care NHS Foundation Trust, Manchester BL9 0EQ, UK; ^2^The University of Manchester, Manchester M13 9PL, UK

## Abstract

Although medical events in lung cancer screening (LCS) such as receiving scan results or interactions with clinicians are recognised as teachable moments (TMs), the views of patients about why this is the case for smoking behaviour change remain uncertain. This systematic review and metasynthesis study is aimed at identifying the reasons why patients believed that medical events during LCS act as TMs for smoking behaviour change. A search strategy was developed for use with MEDLINE, PsycINFO, EMBASE, CINAHL-P, Web of Science databases, and Google Scholar. This helped identify qualitative and mixed-method research which mentioned patients' views of how these TMs result in smoking behaviour change. After screening, final articles were critically appraised; general characteristics and data relevant to the aims were extracted to conduct a line-of-argument metasynthesis. After screening 695 papers, 11 were included. Undergoing LCS scans was seen to act on their intrinsic motivation to reduce smoking as it served as a “wake-up call” and increased awareness of the health consequences of smoking. Receiving positive or negative LCS results resulted in cessation as it was a “health scare” and challenged smoking habits. Interactions with clinicians addressed misconceptions and signposted them to specialist cessation services. Attendees believed that the following encouraged them to change their smoking behaviour: having an intrinsic motivation to quit, their beliefs on smoking and health reframed, their negative emotions appraised, and using LCS to access specialist support. In line with the TM heuristic, these experiences provided the necessary skills, confidence, and motivation to quit. Future research should explore whether the views of the clinicians match those of the attendees to address misconceptions and further develop clinical guidelines.

## 1. Introduction

Around 11.4% of the global population is diagnosed annually with lung cancer, accounting for 18% of all cancer-related deaths [1]. Up to 20% of the global population smokes tobacco [2], which is associated with a lower quality of life, increased comorbidities (e.g., heart diseases [3, 4]), and accounts for two-thirds of lung cancer deaths [1]. Despite implementing global policies to reduce smoking, the addictive aspect of smoking makes cessation difficult [2, 5]. Therefore, research needs to identify how to encourage the public to stop smoking and prevent these outcomes.

Lung cancer screening (LCS) has reduced the prevalence of lung cancer and its mortality rate by 24%-33% [1, 6, 7]. LCS is used to assess high-risk individuals to identify lung cancer at early stages; these can include conversations with clinicians, low-dose computed tomography (LDCT), and support to quit smoking [8]. Researchers determined that attending LCS (i.e., undergoing LDCT scans) [9], receiving a result or diagnosis [10], interacting with clinicians [11], and introducing additional smoking cessation interventions during LCS (e.g., nicotine replacement therapy [12]) are teachable moments (TMs) to facilitate smoking behaviour change. These medical events within LCS were identified as TMs as they are naturally occurring health events where people become suddenly motivated to implement risk-reducing health behaviours [13].

Current research has been conducted quantitatively and therefore fails to consider LCS attendees' views, making it difficult to apply the findings in a person-centred approach within the practice. This gap could be addressed by qualitatively exploring and understanding attendees' views to effectively facilitate and maximise the benefits of these TMs for smoking cessation. Understanding what patients view as TMs is invaluable to healthcare providers because patients explain that they are susceptible to changing their smoking behaviours at specific points within the LCS [14]. By identifying TMs that patients recognise as an opportunity to change and are open to engaging with, healthcare can capitalise on those specific patient-provider interactions in the future. Therefore, acting upon these TMs in practice has the potential to reduce the likelihood and severity of lung cancer, by encouraging patients to quit smoking [12]. This review could inform clinical guidelines around the conduct of LCS to adopt a patient focus and improve its use in practice.

The aim of the systematic review and metasynthesis was to determine LCS attendees' views on why medical events in LCS are TMs for smoking behaviour change.

## 2. Materials and Methods

This review was preregistered on [PROSPERO 2021, CRD42021295545] and followed the PRISMA guidelines to report qualitative systematic reviews [15].

### 2.1. Search Strategy

The search strategy was designed using the SPIDER framework (i.e., sample, phenomenon of interest, design, evaluation, and research type), as it was the most efficient way to identify all relevant articles for this qualitative review [16]. A combination of specific and broad search terms was piloted to maximise the quantity of studies that were eligible for inclusion. For example, it was beneficial to incorporate terms relating to LCS rather than TMs themselves as there were multiple inconsistencies with the terminologies of TMs across studies.  Table 1 denotes the details of the SPIDER search string with the justification of the terms used.

A systematic search was conducted on electronic databases (i.e., MEDLINE, PsycINFO, EMBASE, CINAHL-P, and Web of Science) and grey literature (i.e., Google Scholar) to identify articles up to December 2021 and was updated again in July 2022 to include new research. This updated search did not identify new articles pertaining to the scope of this review. Multifield search builders were used to identify appropriate studies. Searches were not restricted to any attendee demographics, country, and publication dates. Reference lists of included papers were hand-searched to identify potential articles, including forward and backward searching of key papers.

### 2.2. Study Selection

Journal articles were selected if they were published in English, explored intentional or actual smoking behaviour change after LCS, involved attendees' views on the medical events of LCS as TMs, and used mixed or qualitative methods and analysis. Studies were excluded if they focused on interventions/specialist support (e.g., psychoeducation) without involving LCS, pertained to professionals' views without any input from LCS attendees, only provided guidance and/or recommendations for clinical practice, and were published or unpublished thesis, protocols, letters, reviews, commentaries, book chapters, and conference abstracts.

### 2.3. Study Screening

The searched articles were exported and screened on Microsoft Excel, where duplicate articles were automatically and manually removed. Two researchers manually and independently screened titles, abstracts, and full articles based on the eligibility criteria. Both researchers checked and discussed discrepancies, interceded by the third researcher to reach a consensus.

### 2.4. Data Extraction

The researcher recorded study characteristics and attendees' views about LCS-related TMs which they believed accounted for intentional or actual smoking behaviour change onto a standardised data extraction sheet, which was created a priori. A sample data extraction sheet is denoted in Appendix A.

### 2.5. Quality Assessment

Both researchers assessed and cross-checked the quality of all the final studies using the standardised CASP qualitative checklist ([17]; Table 1). The discrepancies were addressed by the third researcher. Studies received a score ranging between 0 and 10 (1 = “yes,” 0.5 = “cannot tell,” and 0 = “no per question). High-quality papers scored between 9 and 10, moderate-quality papers scored between 7.5 and 9, and scores less than 7.5 were classed as low-quality papers [18]. Regardless of the quality, no papers were excluded from the review.

### 2.6. Data Analysis and Synthesis

Two researchers analysed the data using Noblit et al.'s [19] seven phases of metaethnography, which was verified by a third researcher. Both researchers familiarised themselves with the articles and extracted data based on the categories of the “study findings relevant to aim” which included participant quotes, “research input” which included author interpretations, and “other” sections of the data extraction sheet. They then conducted line-by-line coding of first-order constructs (i.e., participant quotes) and second-order constructs (i.e., author interpretations). They inputted this information into a shared PowerPoint document which served as the codebook. The researchers worked collaboratively to develop third-order constructs (i.e., research teams' interpretation) based on the previous order constructs. Themes emerged through a line-of-argument synthesis to determine relationships between all articles. The translated findings were then synthesised and expressed by the research team to develop new interpretations.

## 3. Results

### 3.1. Study Selection

The systematic search generated 695 papers, with 146 duplicates removed. A further 515 papers were excluded when screening the titles and abstracts against the eligibility criteria. The full texts of the remaining 34 papers were screened, with 25 papers being excluded because they omitted medical events in LCS as TMs (*n* = 15), smoking behaviours (*n* = 4), lung cancer (*n* = 1), or attendees' views on smoking (*n* = 5).

**Figure 1:**
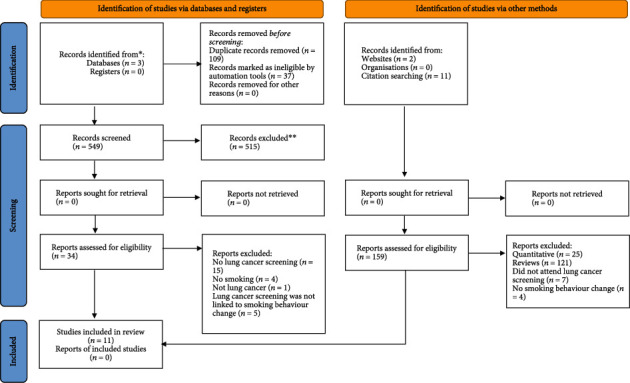
PRISMA flowchart showing the process of study identification.

One paper was identified from the first 100 searches on Google Scholar (i.e., grey literature), and another was selected from the forward and backward searching of the ten remaining papers. Eleven papers were included in this final stage of the review. The PRISMA flowchart depicting the search and selection processes is outlined in Figure 1.

### 3.2. Quality Appraisal

The studies included were of mixed quality, whereby nine were of moderate quality and two were of high quality. The appropriateness of the research design to address the aim and value of the research was the hardest to determine. Most papers did not specify the relationship between researchers and participants. Details of the quality appraisal are outlined in Table 2.

### 3.3. Study Characteristics

All studies involved participants who attended LCS regardless of their test result, except for two studies that included patients with positive results. All studies involved the views of patients who formerly or currently smoke except for one study, which included only patients who currently smoke. Attending LCS scans (*n* = 7), receiving a result or diagnosis (*n* = 10), and patient-professional discussions (*n* = 9) were identified as TMs by the articles. All studies used qualitative semistructured interviews, whereby nine were individual interviews and two used focus groups. Data from these studies were analysed using content analysis (*n* = 6), framework analysis (*n* = 2), thematic analysis (*n* = 1), interpretative phenomenological analysis (*n* = 1), and a systematic iterative analytic process (*n* = 1). These studies were conducted between 2009 and 2020 in either the USA (*n* = 8) or the UK (*n* = 3). Further details of the general characteristics of these papers are described in Table 3.

### 3.4. Metasynthesis

**Figure 2:**
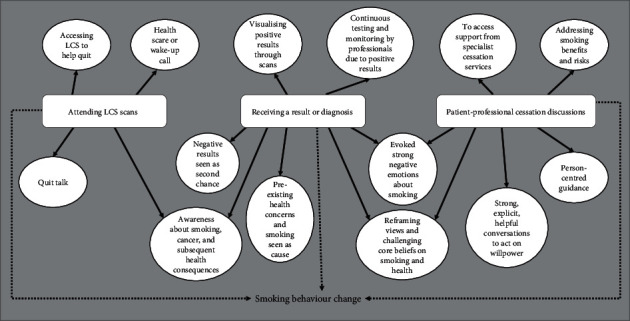
Conceptual map depicting reasons attendees believed that each medical event in lung cancer screening was a teachable moment for smoking behaviour change. Note: three key themes (attending lung cancer screening scans, receiving a result or diagnosis, and patient-professional cessation discussions) were depicted, presented in white rectangles. From these themes, arrows point to white circles containing common reasons why that medical event is believed to be a teachable moment for the attendees. An arrow with a dotted line from each of the key three themes points to smoking behaviour change. LCS = lung cancer screening.

From synthesising 11 studies, 3 key themes were created which centre around medical events during LCS. These themes explain how attendees view specific medical events as TMs to encourage intentional or actual smoking behaviour change (see Figure 2).

#### 3.4.1. Theme 1: Attending LCS Scans

Zeliadt et al. [30] showed that “three out of 37” participants had successfully stopped smoking, whereas the remaining studies found that attending LCS alone (i.e., LDCT scans) only resulted in intentional quitting or reduced smoking through “quit talk” [21, 25, 28]. Some participants felt LCS occurred at the right time and were sufficient to serve as “a wake-up call” or a “health scare” to quit smoking as it made participants “more cognizant of their smoking behaviours and potential health consequences of smoking” [21, 25, 30, 31]. Some participants pursued LCS solely to attempt smoking cessation [21, 28, 30]. They thought that the combination of LCS and other nonscreening factors (e.g., social pressures) instigated smoking behaviour change [28, 32].

Yet, some did not think attending LCS scans acted as a TM for smoking behaviour change. Although they knew it was time to quit smoking, participants lacked interest and did not feel sufficiently motivated by LCS to change their intentions or behaviours [24, 28, 32]. They did not have the urgency to stop smoking [32] and “lacked meaningful connections between cancer, smoking, and health” [25, 28, 29].

The research team interpreted that the process of attending LCS and undergoing LDCT facilitated an intention to quit or reduce smoking but was insufficient for complete cessation as LCS did not raise awareness about lung cancer, smoking habits, and subsequent health consequences. Patients may have had some intrinsic motivation prior to LCS, and the process of attending LCS scans strengthened their intention to quit. It is likely that the combination of attending LDCT scans and experiencing other non-screening factors are needed to increase motivation to reduce or quit smoking.

#### 3.4.2. Theme 2: Receiving a Result or Diagnosis

Participants who received negative results felt they had a “second chance” to mitigate future cancer risks and take care of themselves by cutting down [21, 23] or stopping smoking [28, 30]. However, some participants deemed negative or relatively clear results to be insufficient for smoking behaviour change because it was misunderstood as “permission” to continue smoking, and some mentioned quitting if they received positive results in the future [23, 24, 27, 28].

Mishra et al. [24] showed that “five out of 22 participants” quit smoking due to a “major scare (e.g., receiving test results about a suspicious lung nodule).” Incidental (i.e., unexpected) findings led to smoking reduction while those with indeterminate (i.e., inconclusive) results were more likely to adopt complete cessation [22, 24]. Patients were more likely to quit if they believed smoking was the cause [26], had preexisting health concerns [22], or had the “willpower” to quit [29].

Mishra et al. [24] found that “three out of 22 participants” quit smoking because of their positive results. Positive results increased motivation to quit smoking because they were prompted to think about their smoking habits and were regularly monitored by professionals to avoid worsening their lung cancer [21, 28, 30]. Some participants felt more determined towards smoking cessation when they visualised positive results through scans [21, 24]. Participants who received positive results felt strong negative emotions such as anxiety, shock, surprise, and guilt, which internally motivated them to consider smoking cessation and stay abstinent [21, 23, 28]. Thus, taking advantage of emotional responses from LCS can lead to smoking behaviour change [22]. Yet, some participants with positive results continued smoking because they had “fatalistic” views around smoking [24, 25], failed to have strong negative emotions [21], wanted to wait for further testing, or did not understand the severity or meaning of positive results [23, 28, 30].

The research team deduced that the process of patients receiving a result, regardless of the type of result, can facilitate smoking reduction or cessation. The degree of smoking behaviour change is likely to depend upon their knowledge, reasoning, and interpretation of their results, especially if it challenges their values around smoking. This third-order construct was also corroborated by two studies which claimed that using a person-centred approach and acting upon other motivating factors (e.g., “health concerns”) while delivering results can address core beliefs around smoking to instigate behaviour change [22, 28].

#### 3.4.3. Theme 3: Patient-Professional Smoking Cessation Discussions

Some participants thought that the current approach was ineffective [32], while most explained that they completely lacked these discussions [21, 29]. However, when discussions occurred, a few were not swayed by cessation conversations [32] while others were reluctant to engage in them because of the stigma and guilt associated with smoking [27]. Only “two of the 33 individuals” were inclined to quit smoking because of their discussions with clinicians [32] because clinicians rarely reframed views about smoking and did not link LCS to smoking [21, 22, 30, 32].

Participants wished clinicians gave “explicit,” “strong,” and “helpful” messages to keep them motivated to quit [21, 27–29]. Zeliadt et al. [30] described a participant who felt motivated to quit when their clinician was persistent and “badgered” them while a few participants were deterred from quitting because they felt pushed by clinicians [32]. To encourage smoking behaviour change, participants believed clinicians needed to be proactive and sensitive about tackling smoking [29].

A few participants experienced short-lived emotions such as “shock,” where they quit smoking temporarily but relapsed when they did not see smoking as a threat [27]. It was likely that these emotions were temporary as participants received minimal input from clinicians about smoking cessation during LCS [29, 32].

Some participants wanted extra support to quit smoking but did not receive any guidance from clinicians [27]. For abstinence, clinicians need to continually work collaboratively with patients to provide 1 : 1 smoking cessation discussions and support for relapse prevention [24, 27]. Patients were more receptive to stop smoking when clinicians made them feel more comfortable, addressed the benefits and risks of smoking, and provided clear guidance and referrals to smoking cessation services as a part of their treatment plan [24, 26, 27, 29]. “Three of the 14” participants stayed abstinent with the help of smoking cessation groups, smoking cessation advisers, and community pharmacists because they believed these specialists were competent to address smoking habits and give guidance based on individual needs for cessation [28]. The authors also highlighted the high quality and “effective interpersonal skills of smoking cessation facilitators” to instigate smoking behaviour change [28]. Despite this, one patient expressed that LCS was a time-consuming process, so they did not feel that they had the time to engage with smoking cessation services [28]. Another patient felt there was a lack of privacy when cessation information was delivered in pharmacies and thought generic “stop smoking” messages were not appropriate for those who had an established lung cancer diagnosis [28]. A few patients conveyed that clinicians hardly provided timely information about the cessation or direct referrals to cessation services [28]. Others thought they could quit smoking through “willpower” and viewed nicotine replacement therapy and behavioural support as ineffective [27–29].

The research team concluded that cessation discussions with clinicians could potentially be an effective factor to instigate quit intentions, reduced smoking, or sustained cessation. Most participants wish discussions encompassed strong messages, nonjudgemental approaches, and active discussions about smoking. Thus, clinicians need to adopt an individualised approach to externally motivate smoking behaviour change, link LCS to smoking behaviours, and reframe beliefs about smoking. To encourage long-term cessation, providers should disseminate information about cessation services and streamline referral processes to these services during LCS to ensure that behaviour change is maintained.

### 3.5. Line-of-Argument Synthesis

**Figure 3:**
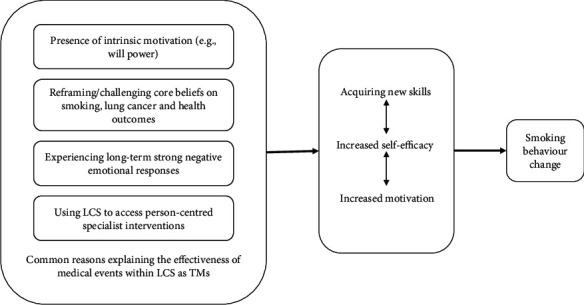
Conceptual map of the line-of-argument depicting the main reasons attendees believed that medical events in lung cancer screening were teachable moments for smoking behaviour change in line with the teachable moment heuristic ([13]). Note: on the far left is a box labelled “common reasons explaining the effectiveness of medical events within lung cancer screening as teachable moments which contains the four main reasons across all teachable moments that attendees” believed to lead to smoking behaviour change. From this box, an arrow points right to a smaller box, which depicts how these four reasons help attendees acquire new skills, increase self-efficacies, and increase motivation. From this box, another arrow points right to the smallest box, representing how this results in smoking behaviour change. LCS = lung cancer screening.

Attendees believe that the process of undergoing LCS scans results in intentional quitting or smoking reduction while receiving a result or lung cancer diagnosis and having discussions with professionals are TMs to cause actual smoking reduction or cessation. However, some attendees identified four reasons across all TMs which they believed were most impactful to result in smoking behaviour change (see Figure 3). Firstly, attendees felt that they needed to have some intrinsic motivation to quit (e.g., willpower). Secondly, they thought their core beliefs around smoking needed to be reframed or challenged, regardless of their results. Thirdly, they felt they needed to encounter long-term strong negative emotional responses (e.g., shock, anxiety, and feelings of urgency) for cessation. Finally, they believed that they could use LCS to access person-centred specialist interventions for cessation and relapse prevention. In the absence of these experiences or interpretations, the likelihood of patients adopting smoking behaviour change decreases.

## 4. Discussion

The aim of this review was to determine LCS attendees' views on why medical events in LCS act as TMs [13] for smoking behaviour change. Patients felt that attending LCS (i.e., LDCT scans), receiving results or a lung cancer diagnosis, and having discussions with clinicians were medical events in LCS that can act as TMs to facilitate smoking cessation. They typically stated that these worked for the following reasons: (1) attending LCS (i.e., LDCT scans) was a “wake-up call” and acted on their intrinsic motivation to quit; (2) rather than the results themselves, their interpretation of the LCS results evoked negative emotions and challenged their core beliefs about smoking and health; or (3) clinicians could effectively manage negative emotions, address misconceptions around results, and signpost them to specialist cessation support to change their views on smoking. Very few participants identified other less common yet relevant medical events as TMs which suggest that TMs are specific to each person based on their knowledge, attitudes, and beliefs. Thus, professionals need to adopt a personalised approach within LCS to enhance cessation rates. Recognising TMs within LCS can be complex and nuanced. Hence, raising awareness of the most frequently experienced TMs can help professionals identify and capitalise on these medical events to maximise the chances of successful cessation which can also help patients feel adequately supported.

The results of this review are supported by the TM theory by McBride et al. [13], which suggests that having negative emotional responses (e.g., anxiety and shock), understanding perceived risks/benefits of smoking, and redefining oneself (e.g., challenging core beliefs and reframing views on smoking and health) are needed for cessation. Thus, experiencing these medical events as TMs encourages them to acquire new skills to cope (e.g., managing cravings), increases their self-efficacies, and further motivates them for smoking behaviour change [13]. The extent of these TMs to elicit smoking behaviour change varied. Attending LCS scans was likely to instigate intention to quit and actual smoking-reducing behaviours without complete cessation, while the remaining TMs were more likely to promote actual smoking reduction or cessation.

These findings were also corroborated by previous studies. The process of attending LCS scans was a TM for patients to decide if they wanted to quit, suggesting that attending appointments acted upon their intrinsic motivation to quit [12, 33, 34]. Receiving LCS results acts as a TM as it challenges their views on smoking habits, leading to behaviour change [35, 36]. The presence of strong negative emotions (e.g., anxiety) and prior health concerns was effective to reduce smoking or cause cessation [9, 37–39]. Studies have suggested that current guidelines for patient-clinician discussions (i.e., conversations about results) are inadequate to change smoking behaviours [20, 40]. However, when coupled with specialist interventions (e.g., telephone counselling) or intensive and personalised cessation advice, they were more likely to quit [12, 20, 40]. This suggests that delivering effective discussions can instigate behaviour change.

This review was one of the first to qualitatively determine the reasons why patients believed that medical events in LCS act as TMs for smoking behaviour change, which could not be identified through quantitative research. The studies reported in this review only included clinical populations from the US and UK, which may have occurred because these countries have established screening processes which specifically address smoking behaviours or have specific staff roles such as cessation nurses [41, 42]. There is also the possibility that different countries use different terminologies for LCS which may have been omitted in the search terms of this review, which warrants further research. Using a metasynthesis within this review was advantageous as it offered rich interpretations of higher-order constructs to effectively address the aim and helped in synthesising findings to generate recommendations that can be applied to improve current practice [43–45]. Although the 11 studies were of moderate to high quality, it was difficult to determine the research value and participant-author relationship because of the lack of information described in the studies, suggesting that their quality could have been higher than what was reported [46]. Although the authors claimed the application of their findings was limited, this metasynthesis showcased the similarities and differences across studies, which expanded the generalisability of the findings.

Although participants described several reasons, very few undertook actual smoking reduction and cessation which suggests the need to specify recommendations and improve clinical practice to capitalise these TMs. Although clinical guidelines [41, 42] recommend that providers should adopt a person-centred approach to maximise TMs for smoking behaviour change, participants reported that this has been difficult to receive in practice. Furthermore, as knowledge and attitudes vary across patients, it can be extremely challenging for professionals to find the balance between sharing technical information and focusing on emotions in a time-sensitive manner. Cessation discussions could be enhanced by standardising the use of communication models such as the 5As (ask, advise, assess, assist, and arrange) with joint decision-making within LCS [20, 32]. Professionals should ensure that discussions remain proactive, collaborative, and sensitive while using explicit, strong, but helpful messages. Clinicians should provide clear written guidance about smoking cessation, address negative emotional responses evoked during LCS, and correct common misconceptions (i.e., interpretation of results, smoking habits, and health consequences) to help challenge and reframe views about smoking. Services should consider supporting professionals to confidently hold these discussions by offering evidence-based training to identify TMs and strengthen their communication skills. Professionals should refer patients to specialist smoking cessation services, or providers could consider incorporating specialist cessation support within LCS processes, whereby cessation specialists and physicians simultaneously work together to effectively capitalise from these TMs for cessation.

Yet, further research is essential to investigate the effectiveness of these findings and determine if the recommendations suggested improve LCS processes and promote health behaviour change. Future research should also explore whether the views of clinicians match those of attendees to address misconceptions, identify helpful strategies to navigate or enhance TMs within LCS, and further develop clinical guidelines. It would also be beneficial to determine the impact of other TMs alongside medical events in LCS for smoking behaviour change. This would help to further incorporate recommendations into clinical practice and enhance the effectiveness of these TMs. This type of review could be adapted and applied in related fields, for example, investigating patient and clinician views on TMs in screening processes in other cancer populations (e.g., prostate and breast) to identify the extent of modifying health behaviours (e.g., smoking, diet, and sleep) and subsequently improve screening.

## 5. Conclusions

Evidently, medical events in LCS act as a TM for smoking behaviour change. The process of attending LCS (i.e., LDCT scans) encourages attendees to act on their intrinsic motivation to reduce smoking. Participants' interpretation of their LCS results and the discussions they had with clinicians motivated them to adopt smoking cessation as this allowed them to reframe their beliefs around smoking. These events also evoked strong negative emotional responses and motivated them to access person-centred specialist interventions which encouraged cessation and relapse prevention. Future studies should test the effectiveness of the recommendations and identify if these views are also held by clinicians.

## Figures and Tables

**Table 1:** String of search terms.

SPIDER framework	Search string	Justification
Sample	diagnosed with lung cancer OR lung cancer diagnosis OR lung cancer OR lung neoplasm^∗^ OR bronchogenic carcinoma OR lung tumo?r^∗^ OR lung carcinoma AND patient^∗^ OR attendee^∗^ OR at-risk	Different diagnoses were used to identify articles relating to lung cancer attendees and patients
Phenomenon of interest	lung cancer screen^∗^ OR lung cancer screening test^∗^ OR early detection or low dose CT scan OR low dose computed tomography OR LDCT OR early diagnosis OR screen^∗^ OR lung screen^∗^ OR chest x-ray AND smoking cessation OR quit smoking OR stop smoking OR give up smoking OR smoking behavio?r OR reduc^∗^ smoking OR low^∗^ smoking OR decrease^∗^ smoking	Processes involved in lung cancer screening and smoking behaviours were used instead of teachable moments as articles described and named each teachable moment differently
Design	focus group^∗^ OR interview^∗^ OR thematic analysis OR framework analysis OR content analysis OR interpretative phenomenological analysis	The most frequently used qualitative design and analysis methods in this field were used to yield sufficient results
Evaluation	view^∗^ OR belief^∗^ OR thought^∗^ OR feeling^∗^ OR attitude^∗^ OR experience^∗^ OR perception^∗^	These terms ensured that attendees' reasoning was captured
Research type	Qualitative OR mixed-method^∗^	These terms were used to find qualitative aspects of studies

**Table 2:** Quality appraisal of reviewed studies.

Criteria	Golden et al. [20]	Kathuria et al. [21]	Kummer et al. [22]	Meltzer et al. [23]	Mishra et al. [24]	Park et al. [25]	Rowland et al. [26]	Simmons et al. [27]	Wells et al. [28]	Young et al. [29]	Zeliadt et al. [30]
Was there a clear statement of the aims?	1	1	1	1	1	1	0.5	1	1	1	1
Is a qualitative methodology appropriate?	1	1	1	1	1	1	1	1	1	1	1
Was the research design appropriate to address the aims of the research?	0.5	0.5	0.5	1	0.5	0.5	1	1	0.5	1	0.5
Was the recruitment strategy appropriate to the aims of the research?	1	1	1	1	1	1	1	1	1	0.5	1
Was the data collected in a way that addressed the research issue?	1	1	1	1	1	1	1	1	0.5	1	1
Has the relationship between researcher and participants been adequately considered?	0	1	1	0	0	0	0	0	0.5	0	0
Have ethical issues been taken into consideration?	1	1	1	0	1	1	0.5	0	1	0.5	1
Was the data analysis sufficiently rigorous?	1	1	1	0.5	1	1	1	1	0.5	1	1
Is there a clear statement of findings?	1	1	1	1	1	1	1	1	1	1	1
How valuable is the research?	0.5	0.5	0.5	1	1	0.5	0.5	1	0.5	1	0
Total	8	9	9	7.5	8.5	8	7.5	8	7.5	8	7.5

**Table 3:** Brief characteristics of the analysed papers.

Study	Aim(s)	Setting	Sample characteristics	Outcomes measured	Study design	Main findings
Golden et al. [20]	To evaluate the experiences of patients who formerly or currently smoke who underwent LCS decision-making discussions.	USA	33 patients who currently smoke and 18 patients who formerly smoke. LDCT results were not recorded.	Patients' views on patient-clinician interactions.	Qualitative 1 : 1 semistructured interviews.	(1) LCS decision-making discussions rarely influenced smoking behaviour change. (ii) Increased reframing elicits negative emotional responses, making discussions an effective TM.

Kathuria et al. [21]	To understand patients' views on communication about LCS and smoking cessation, integration of smoking cessation discussions within LCS, and if LCS discussions are a TM for smoking cessation.	USA	28 patients who currently smoke and 21 patients who formerly smoke. LDCT results were not recorded.	Patients' views on patient-clinician interactions.	Qualitative 1 : 1 semistructured interviews and focus groups.	(i) LCS was a TM for smoking cessation because LCS prompted awareness of the harms of smoking and evoked vulnerability, relief, and worry. (ii) Patients had varied experiences of LCS discussions because they viewed the purpose of LCS differently from one another.

Kummer et al. [22]	To explore positive and negative psychological and behavioural responses among individuals with indeterminate (i.e., unclear) and incidental (i.e., discovered by chance) LDCT screening results.	USA	18 patients who currently smoke and 10 patients who formerly smoke. LDCT results: indeterminate (*n* = 10), incidental (*n* = 13), and negative (*n* = 5).	Patients' views on LCS pathway (i.e., referral, communication, and result).	Qualitative 1 : 1 semistructured interviews.	(i) The LDCT result, concerns and health expectations, negative beliefs, and perceived stigma influenced individual differences in psychological and behavioural responses. (ii) Patients with incidental results cut down on smoking when compared to patients with indeterminate results.

Meltzer et al. [23]	To develop and examine the feasibility and acceptability of a self-help smoking cessation intervention when patients who smoke viewed low-dose computed tomography (LDCT) to be a teachable moment.	USA	31 patients who currently smoke LDCT results: positive (*n* = 11) and negative (*n* = 20).	Patients' views on LCS with LDCT and smoking cessation via focus groups.	Focus groups (*n* = 15) and learner verification interviews (*n* = 16) using semistructured interview guides.	(i) Focus group participants wished the process of receiving LDCT addressed counterproductive thoughts about negative LCS results. (ii) They wanted to enjoy a healthy and smoke-free retirement, increase self-efficacy about smoking cessation, and see survival statistics after quitting. (iii) Learner verification participants favoured most booklet and pamphlet changes. (iv) Feasibility findings showed high acceptability and satisfaction of the LDCT self-help cessation intervention.

Mishra et al. [24]	To determine patients' knowledge and attitudes about LDCT, LCS, smoking cessation, and decision-making for LCS.	USA	Nine patients who currently smoke and 13 patients who formerly smoke. LDCT results: positive (*n* = 12) and negative (*n* = 10).	Patients' views on LCS with LDCT and smoking cessation.	Qualitative 1 : 1 semistructured interviews.	(i) Many patients were unaware of LDCT but were open to engaging in LCS. (ii) Some contemplated quitting if they received positive results. (iii) Patients preferred 1 : 1 discussion with clinicians during LCS to support decision-making.

Rowland et al. [26]	To explore the health-related quality of life (HRQoL) and support experiences among newly diagnosed patients with advanced lung cancer.	UK	Three patients who currently smoke and six patients who formerly smoke. All participants had a positive diagnosis of lung cancer.	Patients' views on LCS and smoking cessation.	Qualitative 1 : 1 semistructured interviews.	(i) Patients feared compromising their immune systems and adjusting to new relationships which impacted their HRQoL. (ii) Patients knew about the links between lung cancer and smoking but continued smoking. (iii) Those who recently quit or continued smoking were sensitive to the opinions of medical staff about smoking.

Park et al. [25]	To explore if LCS or risk perceptions act as a cue for smoking behaviour change and identify postscreening behavioural intentions and changes.	USA	17 patients who currently smoke and 18 patients who formerly smoke. LDCT results: true positive (*n* = 1), false positive (*n* = 10), and negative (*n* = 24).	Patients' views on smoking behaviour following LCS.	Qualitative 1 : 1 semistructured interviews.	(i) High-risk perceptions and low confidence in quitting did not translate to cessation after LCS. (ii) Cognitive, emotional dissonance, and avoidance strategies deterred smoking behaviour change.

Simmons et al. [27]	To fill a gap in research by examining cancer patient-provider communication regarding tobacco use and patients' perspectives regarding their experiences with smoking cessation and relapse.	USA	10 patients who currently smoke and 10 patients who formerly smoke. All participants had a positive diagnosis of lung cancer.	Patients' views on LCS and smoking cessation.	Qualitative telephone-based 1 : 1 semistructured interviews.	(i) They had high motivation to quit smoking but did not ask for assistance to quit and maintain abstinence. (ii) Patients who relapsed were reluctant to disclose smoking behaviour due to stigma and guilt. (iii) Patients believed clinicians gave different information, advice, and assistance. (iv) Clinicians asserted the long-term risks of smoking and briefly recommended cessation interventions.

Wells et al. [28]	To explore the experiences and views of patients, family members, and healthcare professionals towards smoking and smoking cessation around the time of cancer diagnosis.	UK	15 patients who currently smoke and 14 patients who formerly smoke. All participants had a positive diagnosis of lung cancer.	Patients' views on LCS and smoking cessation.	Qualitative 1 : 1 semistructured interviews.	(i) Few had meaningful discussions with professionals about smoking. (ii) Some continued to smoke because of the stress after diagnosis and did not understand the link between smoking, cancer, and health.

Young et al. [29]	To understand how lung cancer screening influences individual motivations about smoking, including in those who have stopped smoking since the screening.	UK	20 patients who currently smoke and 11 patients who formerly smoke. Early LDCT results: positive (*n* = 13) and negative (*n* = 18).	Patients' views on LCS with LDCT and smoking cessation.	Qualitative 1 : 1 semistructured interviews.	(i) Interpretation of results led to emotional responses causing smoking behaviour change. (ii) LCS and family support were a “wake-up call” for cessation. (iii) No clear pattern in smoking motivations based on LCS results. (iv) Some underwent screening to try to stop smoking, while others had minimal or no desire to stop.

Zeliadt et al. [30]	To understand views on smoking cessation from patients who formerly or currently smoke when offered LCS.	USA	34 patients who currently smoke and three patients who formerly smoke. LDCT results were not recorded.	Patients' views on LCS within routine care and smoking cessation.	Qualitative telephone-based 1 : 1 semistructured interviews.	(i) LCS prompted patients who currently smoke to reflect on their health which provides an opportunity for patients to engage in cessation discussions. (ii) Discussions should focus on emotional responses to LCS rather than clinical details.

Note: LCS = lung cancer screening; LDCT = low-dose computed tomography; TM = teachable moment; HRQoL = health-related quality of life.

## Data Availability

The qualitative data supporting this systematic review and metasynthesis study are from the previously reported studies and datasets, which have been cited. The processed data are available from the corresponding authors upon request.

## Supplementary Materials

Supplementary Materials Appendix A: data extraction sheet.
