# Supplementary material for: Patients' Views on Medical Events in Lung Cancer Screening as Teachable Moments for Smoking Behaviour Change: A Systematic Review and Metasynthesis
Source: J Smok Cessat. 2023 Jun 3;2023:6647364. doi: 10.1155/2023/6647364 (PMC10257552; doi:10.1155/2023/6647364)
Supplement: Supplementary Materials — Appendix A: data extraction sheet. [file 6647364.f1.docx]

## Supplementary Materials

**Appendix A:** Data Extraction Sheet.

| **Categories** | **Features** |
| --- | --- |
| Article characteristics | Title  Authors  Year  Site/Location |
| Participants | Participant characteristics (number, demographics, smoking history)  Smoking behaviours (e.g., intentional, or actual cessation, reduction, relapse, no change) after or during LCS |
| Study information | Research aims  Data collection method  Data analysis method |
| Study findings relevant to aim | LCS-related TMs  Participants’ views (i.e., quotations or summaries of key messages from participants) |
| Research input | Researcher characteristics  Researchers' views |
| Other | Practical issues  Other identified information |
|  | DOI/ URL |

*Note*. Points in brackets indicate prompts for data extraction
